# Analysis and Fate of Emerging Pollutants during Water Treatment

**DOI:** 10.1155/2013/256956

**Published:** 2013-05-16

**Authors:** Fei Qi, Guang-Guo Ying, Kaimin Shih, Jolanta Kumirska, Elif Pehlivanoglu-Mantas, Xiaojun Luo

**Affiliations:** ^1^Beijing Key Lab for Source Control Technology of Water Pollution, College of Environmental Science and Engineering, Beijing Forestry University, No. 35 Qinghua East Road, Beijing 100083, China; ^2^State Key Laboratory of Organic Geochemistry, Guangzhou Institute of Geochemistry, Chinese Academy of Sciences, Guangzhou 510640, China; ^3^Department of Civil Engineering, The University of Hong Kong, Pokfulam Road, Hong Kong; ^4^Department of Environmental Analysis, Faculty of Chemistry, University of Gdansk, Sobieskiego 18/19, 80-952 Gdansk, Poland; ^5^Environmental Engineering Department, Istanbul Technical University, Ayazaga Campus, Maslak, 34469 İstanbul, Turkey

Emerging pollutants defined as compounds that are not currently covered by existing water-quality regulations all over the world, have not been studied widely before, and are thought to be potential threats to environmental ecosystems and human health. This special issue compiles 5 exciting papers, which are very meticulously performed researches. 

Generally, emerging pollutants encompass a diverse group of compounds, including pharmaceuticals, drugs of abuse, personal-care products (PCPs), steroids and hormones, surfactants, perfluorinated compounds (PFCs), flame retardants, industrial additives and agents, gasoline additives, new disinfection byproducts (DBPs), nanomaterials, and the toxic minerals.

The analysis methods, occurrence, and fate of hormonal and endocrine disruptors compounds (EDCs) were discussed in two papers of this special issue. R. Guedes-Alonso et al. determine the hormonal residues in treated water by ultrahigh performance liquid chromatography-tandem mass spectrometry (UPLC-MS) and evaluate the efficiency of the conventional wastewater treatment for the removal of hormonal compounds. Moreover, Y. Liu et al. study another kinds of PPCPs, named as phthalate esters that is typical kind of EDCs. The occurrence in a surface water and the removal efficiency in a traditional drinking water treatment palnt are studied. According to results of the two papers, the occurrence and fate of hormonal and EDCs in water or wastewater treatment are very clear. 

J. Xing et al. report a new wastewater treatment technology, bioflocculation, for the removal of sulfamethoxazole that is a typical pharmaceutical in wastewater. The performance and the reaction mechanism of the biodegradation of sulfamethoxazole by the bioflocculation are discussed in depth. Moreover, the optimum reaction condition is obtained. 

The heavy metal and toxic mineral are also important pollutants in the environment, especially in the mining area. Two papers of this issue are focused on this. K. Naeemullah et al. report a green preconcentration method for the determination of cobalt and lead in water. Y. Liu et al. do a novel research on the electrochemical reaction of pyrite as a simulation of the natural environmental.

By compiling this special issue, we hope to enrich our readers and researchers on the analysis and fate of emerging pollutants during water treatment.

*Fei Qi* *Fei Qi* *Guang-Guo Ying* *Guang-Guo Ying* *Kaimin Shih* *Kaimin Shih* *Jolanta Kumirska* *Jolanta Kumirska* *Elif Pehlivanoglu-Mantas* *Elif Pehlivanoglu-Mantas* *Xiaojun Luo* *Xiaojun Luo*

